# Alterations of pulmonary vascular afterload in exercise‐induced pre‐ and post‐capillary pulmonary hypertension

**DOI:** 10.14814/phy2.15559

**Published:** 2023-01-12

**Authors:** Elizabeth Karvasarski, Robert F. Bentley, Tayler A. Buchan, Felipe H. Valle, Stephen P. Wright, Isaac S. Chang, John T. Granton, Susanna Mak

**Affiliations:** ^1^ Sinai Health/University Health Network Toronto Ontario Canada; ^2^ Institute of Medical Science, Faculty of Medicine University of Toronto Toronto Ontario Canada; ^3^ Faculty of Kinesiology and Physical Education University of Toronto Toronto Ontario Canada; ^4^ University Health Network Toronto Ontario Canada; ^5^ Department of Medicine University of Toronto Toronto Ontario Canada; ^6^ Hospital de Clínicas de Porto Alegre Porto Alegre Brazil; ^7^ Heart and Vascular Institute University of British Columbia Kelowna British Columbia Canada

**Keywords:** exercise, hemodynamics, pulmonary hypertension, resistance‐compliance time

## Abstract

Exercise imposes increased pulmonary vascular afterload based on rises in pulmonary artery (PA) wedge pressure, declines in PA compliance, and resistance‐compliance time. In health, afterload stress stabilizes during steady‐state exercise. Our objective was to examine alterations of these exercise‐associated stresses in states of pre‐ and post‐capillary pulmonary hypertension (PH). PA hemodynamics were evaluated at rest, 2 and 7 min of steady‐state exercise at moderate intensity in patients who exhibited Pre‐capillary (*n* = 22) and post‐capillary PH (*n* = 22). Patients with normal exercise hemodynamics (NOR‐HD) (*n* = 32) were also studied. During exercise in all groups, PA wedge pressure increased at 2 min, with no further change at 7 min. In post‐capillary PH and NOR‐HD, increases in PA diastolic pressure and diastolic pressure gradient remained stable at 2 and 7 min of exercise, while in pre‐capillary PH, both continued to increase at 7 min. The behavior of the diastolic pressure gradient was linearly related to the duration of resistance‐compliance time at rest (*r* ^2^ = 0.843) and exercise (*r* ^2^ = 0.760). Exercise resistance‐compliance time was longer in pre‐capillary PH associated with larger increases in diastolic pressure gradient. Conversely, resistance‐compliance time was shortest in post‐capillary PH compared to pre‐capillary PH and NOR‐HD and associated with limited increases in exercise diastolic pressure gradient. During steady‐state, modest‐intensity exercise‐specific patterns of pulmonary vascular afterload responses were observed in pre‐ and post‐capillary PH relative to NOR‐HD. Longer resistance‐compliance time related to greater increases in PA diastolic pressure and diastolic pressure gradients in pre‐capillary PH, while shorter resistance‐compliance time appeared to limit these increases in post‐capillary PH.

## Graphical abstract

We assessed patterns of change in hemodynamics and pulmonary vascular afterload over a period of steady‐state exercise in patients with pre‐ and post‐capillary pulmonary hypertension. Patients with normal hemodynamics were also studied. The responses of the resistance‐compliance time, prolonged in pre‐capillary pulmonary hypertension and shortest in post‐capillary pulmonary hypertension, were strongly associated with the diastolic pressure gradient. These differences accounted for the distinctive patterns of change in pulmonary vascular afterload observed between pre‐ and post‐capillary pulmonary hypertension.

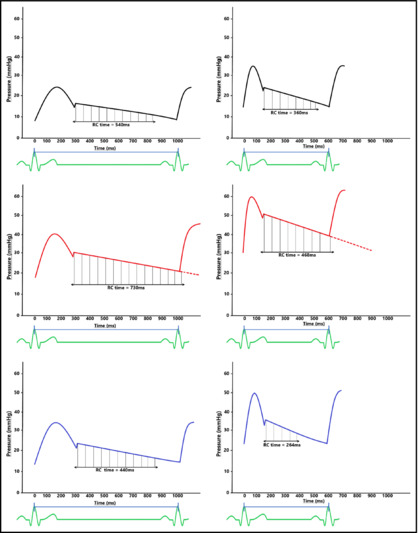

## 1 INTRODUCTION

Exercise presents a complex physiologic challenge to pulmonary arterial and pulmonary venous circulation. In healthy volunteers, at the onset of exercise, we observed a rapid increase in pulmonary artery wedge pressure (PAWP) followed by stabilization as exercise continued at a constant work rate, over several minutes (Wright, Esfandiari, et al., 2016). We also demonstrated that exercise was associated with increases in the pulsatile component of pulmonary vascular afterload (manifested as declines in the pulmonary artery compliance, PAC) relative to modest decreases in the resistive component (pulmonary vascular resistance, PVR). This results in a shortening of the resistance‐compliance (RC) time product (Wright, Granton, et al., 2016). The response of pulmonary artery pressures (PAP) and other pulmonary vascular measurements closely paralleled the PAWP, such that all changes occurred rapidly and then stabilized during exercise at a constant work rate (Wright, Granton, et al., 2016).

If the PAWP is the stimulus for change in PAC and RC time in health, these relationships are likely quite deranged in patients with pulmonary hypertension (PH), with possible disparate responses between pre‐capillary (Pre‐cap) versus post‐capillary (Post‐cap) PH. As the duration of RC time also reflects the duration of diastolic PAP decay, the relative length of RC time may also influence the temporal pattern of pulmonary vascular behavior as exercise increases heart rate (HR) and shortens the RR interval. As such, PAP may not stabilize during constant work‐rate exercise in states of PH, as they do in healthy subjects. Therefore, we tested the hypothesis that PAP will continue to change despite a constant work‐rate exercise in different states of PH. Our study objectives were to compare pulmonary vascular hemodynamics over a period of exercise at a constant work rate between patients with Pre‐cap PH, or Post‐cap PH as well as a group with normal hemodynamics at rest and exercise (NOR‐HD). We also aimed to describe the relationships between PAWP, PAC, PVR, and RC time product and how these responses relate to the changes in PAP over a bout of constant work‐rate exercise.

## 2 METHODS

### 2.1 Hemodynamic registry

Consecutive cases were evaluated from a database of patients with dyspnea and/or suspected PH of uncertain etiology who underwent exercise right‐heart catheterization (RHC) in our laboratory between December 09, 2017 and February 20, 2020.

### 2.2 Cardiac catheterization and exercise protocol

Right‐heart catheterization was performed and hemodynamics were recorded at resting and during exercise conditions as we have previously published (Bentley et al., 2020; Wright, Esfandiari, et al., 2016; Wright, Granton, et al., 2016). Supine hemodynamics were recorded first, then patients were transferred to a cycle ergometer and tilted into a semi‐upright position. Transducers were re‐zeroed at the level of the midaxillary line. Hemodynamics were resampled in the semi‐upright position (rest) prior to initiating a 1 min warm‐up (unloaded pedaling). This analysis included data acquired throughout the first exercise work rate (15–40 Watts). Right atrial pressure (RAP) and PAP were transduced continuously, while PAWP was acquired intermittently with balloon inflation at rest, 2 and 7 min of the exercise stage, similar to our previous work in healthy subjects (Wright, Esfandiari, et al., 2016). Thermodilution CO in triplicate (≤10% variation) was measured at rest and after 2 min of exercise. The workload was determined based on the Medical Research Council (MRC) dyspnea scale. Prior to the study, patients completed the MRC dyspnea scale and two work rates were selected based on the MRC score: MRC >3–15 W, MRC <3–25 W for women, 40 W for men.

### 2.3 Hemodynamic analysis and classifications

Exercise PH classifications were based on pressure flow relationships:

NOR‐HD: mean PAP/cardiac output (mPAP/CO) ≤3 mmHg/L/min.

PH: mPAP/CO > 3 mmHg/L/min.

Pre‐cap PH: mPAP/CO > 3 mmHg/L/min AND the slope of the change in PAWP relative to the change in CO (∆PAWP/∆CO) ≤ 2 mmHg/L/min.

Post‐cap PH: mPAP/CO > 3 mmHg/L/min AND ∆PAWP/∆CO > 2 mmHg/L/min.

As our objective was to contrast pre‐ and post‐capillary PH, cases were excluded if the definition for post‐cap PH was met but pre‐cap PH was also present, defined as the slope of the change in transpulmonary gradient relative to cardiac output (∆TPG/∆CO) > 2 mmHg/L/min (Ho et al., 2020; Zeder et al., 2022). Cases were also excluded if there was an inadequate duration of signals (<7 min) or if interruptions in the PAP recordings were longer than 120 s (e.g., PAWP or saturation measurements obtained). The complete analysis required sufficient recordings of the PAP and PAWP for repeated measurements between rest and exercise.

The PAP and PAWP waveforms for each case were analyzed by an investigator (EK) blinded to participant characteristics. Values for PA systolic (PASP), PA diastolic (PADP), mPAP, and PAWP were reported at rest, 2 and 7 min of exercise; for each condition automated digital analysis of at least 10 consecutive beats were required.

Calculated variables included: TPG = mPAP‐mean PAWP, diastolic pressure gradient (DPG) = PADP‐PAWP, PVR = TPG/CO, PA pulse pressure (PP) = PASP‐PADP, PAC = stroke volume (SV)/PP, and RC time (s) = [TPG/(HR × PP)] × 60. The duration of RC time (msec) was also expressed as a percentage of cycle length or RR interval measured in msec (RC time_(%RR)_).

### 2.4 Statistical analysis

Statistics were completed using SPSS 20 (IBM Corp, Armonk, NY). Normality was assessed quantitatively with a Shapiro–Wilk test. Normally distributed data are presented as mean ± standard deviation (*SD*), otherwise, data are presented as median, interquartile range (Q1–Q3). Between group comparisons of clinical and baseline hemodynamic characteristics were analyzed by one‐way analysis of variance (ANOVA) for continuous variables, and chi‐squared analysis for categorical variables. Hemodynamic parameters were measured at rest, 2 and 7 min of exercise and analyzed using a mixed model, repeated measures ANOVA, with exercise condition as one factor and group as the second factor. Bonferroni correction was applied for post hoc pairwise comparisons. For the repeated measures ANOVA, the assumption of sphericity was met. Anticipating age and resting hemodynamic variables would be different between groups, repeated measures ANCOVA was also performed to adjust for the resting values of the hemodynamic variable of interest as the covariate as well as the effect of age. Adjusted mean values are presented in the accompanying figures while tables demonstrate the raw and unadjusted values.

As we hypothesized that the relative length of RC time influences the temporal pattern, a multivariable analysis was conducted to evaluate significant hemodynamic correlates of RC time selected a priori. Representative variables of the hemodynamic response to exercise were entered into the model and included CO, HR, PAWP, DPG, PADP, and PASP. An alpha level of <0.05 was considered statistically significant.

## 3 RESULTS

### 3.1 Subject characteristics

Of 107 cases identified, as prespecified, 4 were excluded as there was combined pre‐ and post‐capillary PH, and 25 were excluded based on inadequate waveforms, leaving 76 cases for analysis. Based on the pre‐specified exercise hemodynamic classification, we identified 22 with pre‐cap PH, 22 with post‐cap PH, and 32 met the definition for NOR‐HD. Table 1 presents subject characteristics. As expected, patients in the Post‐cap PH group were older than the NOR‐HD group. All groups were similar with respect to the proportion of females (~50%), weight, body mass index, and body surface area. Common conditions associated with group 1 pulmonary arterial hypertension (PAH) and cardiopulmonary comorbidities for heart failure with preserved ejection fraction are listed in Table 2. Medications, including specific PAH therapy, are also included in Table 2. Patients with post‐cap PH were more likely to have had a history of HF and a higher proportion of this group were treated with beta‐blockers, loop diuretics, and spironolactone/eplerenone, particularly compared to the NOR‐HD group. A higher proportion of patients in the pre‐cap PH group were treated with dual PAH therapy than the NOR‐HD group.

**TABLE 1:** Patient demographic and hemodynamic characteristics

Variable	NOR‐HD *n* = 32	Pre‐cap PH *n* = 22	Post‐cap PH *n* = 22
Demographic information			
Sex, % female	47	45	50
Age, years	54 ± 16	62 ± 14	64 ± 12^a^
Height, cm	174 ± 9	171 ± 9	166 ± 8^a^
Weight, kg	86 ± 20	85 ± 23	80 ± 19
BMI, kg/m^2^	29 ± 5	29 ± 7	29 ± 6
BMI >30 kg/m^2^, %	41	50	41
BSA, m^2^	2.0 ± 0.3	2.0 ± 0.3	1.9 ± 0.3
Exercise Classification			
mPAP/CO, mmHg/L/min	2.3 [2.1–2.8]	5.7 [3.8–7.8]	4.8 [3.4–6.8]
∆TPG/∆CO,	0.9 [0.4–1.4]	3.3 [1.5–5.4]	0.2 [−1.1–1.0]
∆PAWP/∆CO	1.2 [0.65–1.8]	0.5 [0.2–0.8]	4.1 [2.3–7.5]
Baseline hemodynamics			
HR, bpm	65 ± 10	67 ± 12	67 ± 7
SV, ml	96 ± 25	76 ± 17^a^	72 ± 21^a^
CO, L/min	6.1 ± 1.6	5.2 ± 1.4	4.8 ± 1.3^a^
SBP, mmHg	130 ± 16	137 ± 23	125 ± 19
DBP, mmHg	75 ± 12	78 ± 12	68 ± 11^b^
MAP, mmHg	93 ± 12	102 ± 20	90 ± 13
RAP, mmHg	4 ± 2	6 ± 3	5 ± 4
PASP, mmHg	27 ± 6	58 ± 22^a^	34 ± 11^a^ ^,^ ^b^
PADP, mmHg	8 ± 3	18 ± 8^a^	12 ± 6^a^ ^,^ ^b^
mPAP, mmHg	17 ± 4	34 ± 13^a^	22 ± 8^a^ ^,^ ^b^
PP, mmHg	19 ± 5	40 ± 16^a^	22 ± 6^b^
mPAWP, mmHg	9 ± 3	9 ± 4	12 ± 6
TPG, mmHg	8 ± 3	25 ± 13^a^	9 ± 5^b^
DPG, mmHg	−1 ± 3.	9 ± 9^a^	0 ± 3^b^
PVR, WU	1.4 ± 0.6	5.0 ± 3.5^a^	1.9 ± 1.3^b^
PAC, mL/mmHg	5.6 ± 2.4	2.3 ± 1.4^a^	3.7 ± 1.8^a^ ^,^ ^b^

*Note*: Data are mean ± *SD* or Median [25th percentile–75th percentile]. Baseline hemodynamics were recorded with patients in the supine position.

Abbreviations: BMI, body mass index; BSA, body surface area; CO, cardiac output; DBP, diastolic blood pressure; DPG, diastolic pressure gradient; HR, heart rate; MAP, mean arterial pressure; mPAP, mean pulmonary artery pressure; mPAWP, mean pulmonary artery wedge pressure; PAC, pulmonary arterial compliance; PADP, pulmonary artery diastolic pressure; PASP, pulmonary artery systolic pressure; PAWP, pulmonary artery wedge pressure; PP, pulmonary artery pulse pressure; PVR, pulmonary vascular resistance; RAP, right atrial pressure; SBP, systolic blood pressure; SV, stroke volume; TPG, transpulmonary gradient.

^a^ *p* < 0.05 vs. NOR‐HD.

^b^ *p* < 0.05 vs. Pre‐cap PH.

**TABLE 2:** Patients comorbidities, medications, and therapies

Variables	NOR‐HD	Pre‐cap PH	Post‐cap PH
Co‐morbidities, *n* (%)			
Connective Tissue Disorder	3 (9)	6 (27)	1 (5)
Idiopathic PAH	0	2 (9)	0
Portal Hypertension	0	1 (5)	0
Diabetes	5 (16)	6 (27)	1 (5)
Hypertension	9 (29)	10 (45)	14 (64)
CAD	7 (22)	6 (27)	7 (32)
Hx of HF	4 (13)	2 (9)	8 (36)^a^
COPD	2 (6)	6 (27)	3 (14)
Documented previous PE	15 (47)	9 (41)	4 (18)
Medications, *n* (%)			
ACE/angiotensin blocker	8 (25)	4 (18)	11 (50)
Beta‐blockers	5 (16)	3 (14)	12 (55)^a^ ^,^ ^b^
Calcium channel blocker	4 (13)	1 (5)	5 (23)
Insulin/Hypoglycemic	5 (16)	3 (14)	1 (5)
Loop diuretic	3 (9)	8 (36)	10 (45)^a^
Spironolactone/eplerenone	2 (6)	4 (18)	6 (27)^a^
Therapy, *n* (%)			
Monotherapy with PDE5i	0	3 (14)	0
Dual therapy with PDE5i and ERA	0	5 (23)^a^	1 (5)
Other combinations	0	2 (9)	0

Abbreviations: ACE, ACE, angiotensin‐converting enzyme; CAD, coronary artery disease; COPD, chronic obstructive pulmonary disease; ERA, Endothelin receptor antagonists; Hx of HF, history of heart failure; PDE5i, Phosphodiesterase 5 inhibitors; PE, pulmonary embolism.

^a^ *p* < 0.05 vs. NOR‐HD.

^b^ *p* < 0.05 vs. Pre‐cap PH.

### 3.2 Hemodynamic characteristics at rest

Hemodynamic measurements in the supine position are presented in Table 1. There were no differences in resting HR between groups. CO was lower in the post‐cap PH group compared to the NOR‐HD group. SV was lower in both the pre‐cap and post‐cap PH groups compared to the NOR‐HD group. At baseline, there were no significant differences in RAP or PAWP between the groups. PASP, PADP, mPAP, and PP were significantly higher in the pre‐cap PH group compared to both the post‐cap PH and NOR‐HD groups.

### 3.3 Pulmonary vascular hemodynamic patterns during steady‐state exercise

Hemodynamics at rest, 2 and 7 min of exercise are presented in Table 3. Exercise work rates were not significantly different between the groups (Table 4). Exercise hemodynamics were significantly different between the groups by design. As expected, increases in HR, CO, SV, RAP, PAPs, and PAWP with exercise were observed in all groups. CO was higher in the No PH group compared to the post‐cap PH group, while SV was higher in the NOR‐HD group compared to both the pre‐cap and post‐cap PH groups during exercise (Table 4). PAWP was highest in the post‐cap PH group. PASP, PADP, and mPAP were highest in the Pre‐cap PH group, intermediate in the post‐cap PH group, and lowest in the NOR‐HD group.

**TABLE 3:** Hemodynamics at rest, 2 and 7 min of exercise at a constant work rate

	NOR‐HD			Pre‐cap PH			Post‐cap PH		
	Rest	2 min exercise	7 min exercise	Rest	2 min exercise	7 min exercise	Rest	2 min exercise	7 min exercise
HR, bpm	68 ± 12	89 ± 14^a^	94 ± 15^a^ ^,^ ^b^	71 ± 10	94 ± 14^a^	102 ± 15^a^ ^,^ ^b^	67 ± 9	85 ± 12^a^	95 ± 13^a^ ^,^ ^b^
RAP, mmHg	2 ± 3	5 ± 4^a^	5 ± 4^a^	3 ± 3	8 ± 6^a^	8 ± 7^a^	3 ± 3	9 ± 6^a^	9 ± 5^a^
PASP, mmHg	23 ± 6	34 ± 9^a^	36 ± 12^a^ ^,^ ^b^	53 ± 23	75 ± 25^a^	83 ± 28^a^ ^,^ ^b^	34 ± 13	49 ± 14^a^	56 ± 18^a^ ^,^ ^b^
PADP, mmHg	8 ± 4	14 ± 5^a^	14 ± 5^a^	18 ± 7	27 ± 9^a^	30 ± 10^a^ ^,^ ^b^	13 ± 8	21 ± 8^a^	22 ± 9^a^
mPAP, mmHg	15 ± 4	23 ± 6^a^	24 ± 7^a^	31 ± 12	47 ± 14^a^	52 ± 17^a^ ^,^ ^b^	22 ± 9	34 ± 9^a^	38 ± 12^a^ ^,^ ^b^
mPAWP, mmHg	7 ± 4	12 ± 5^a^	12 ± 5^a^	7 ± 4	10 ± 4^a^	10 ± 5^a^	11 ± 7	22 ± 8^a^	23 ± 9^a^
PP, mmHg	15 ± 4	20 ± 6^a^	22 ± 8^a^ ^,^ ^b^	35 ± 18	47 ± 17^a^	52 ± 19^a^ ^,^ ^b^	21 ± 8	28 ± 9^a^	34 ± 14^a^ ^,^ ^b^
TPG, mmHg	8 ± 3	11 ± 5^a^	13 ± 5^a^	25 ± 12	37 ± 16^a^	42 ± 19^a^ ^,^ ^b^	11 ± 6	12 ± 9	15 ± 13^a^ ^,^ ^b^
DPG, mmHg	1 ± 3	2 ± 4	3 ± 4	12 ± 8	17 ± 10^a^	21 ± 11^a^ ^,^ ^b^	3 ± 4	−1 ± 7^a^	0 ± 9

*Note*: Data are mean ± *SD*. See Table 1 for abbreviations. Resting and exercise hemodynamics were recorded when the patient was in the semi‐upright position on the cycle ergometer.

^a^ *p* < 0.05 vs. rest.

^b^ *p* < 0.05 vs. 2 min.

**TABLE 4:** Hemodynamics at rest and exercise at a constant work rate

	NOR‐HD		Pre‐cap PH		Post‐cap PH	
	Rest	Exercise	Rest	Exercise	Rest	Exercise
WR, Watts	‐	29 ± 11	‐	23 ± 9	‐	24 ± 8
CO, L/min	5.6 ± 1.2	9.7 ± 2.1^a^	5.0 ± 1.2	8.6 ± 2.9^a^	4.3 ± 1.1^b^	7.3 ± 2.5^a^ ^,^ ^b^
SV, ml	84 ± 19	107 ± 25^a^	69 ± 16^b^	87 ± 24^a^ ^,^ ^b^	64 ± 17^b^	84 ± 30^a^ ^,^ ^b^
CI, L/min/m^2^	2.8 ± 0.6	4.8 ± 1.0^a^	2.2 ± 0.7^b^	4.3 ± 1.2^a^	2.2 ± 0.4^b^	3.8 ± 1.0^a^ ^,^ ^b^
SVi, mL/m^2^	42 ± 9	53 ± 12^a^	35 ± 6^b^	44 ± 9^a^ ^,^ ^b^	33 ± 7^b^	43 ± 12^a^ ^,^ ^b^
PVR, WU	1.4 ± 0.6	1.3 ± 0.6	5.4 ± 3.0^b^	5.9 ± 3.7^a^ ^,^ ^b^	2.9 ± 1.8^b^ ^,^ ^c^	2.5 ± 2.7^c^
PAC, mL/mmHg	6.0 ± 1.8	5.4 ± 2.1^a^	2.6 ± 1.5^b^	2.1 ± 1.5^a^ ^,^ ^b^	3.6 ± 1.9^b^	2.9 ± 1.6^a^ ^,^ ^b^
RC time, s	0.47 ± 0.15	0.38 ± 0.13^a^	0.63 ± 0.18^b^	0.46 ± 0.12^a^	0.50 ± 0.10^c^	0.28 ± 0.11^a^ ^,^ ^b^ ^,^ ^c^
RC time as proportion of RR interval (%)	54 ± 20%	60 ± 25%	73 ± 20%^b^	78 ± 18%^b^	55 ± 11%^c^	44 ± 20%^a^ ^,^ ^b^ ^,^ ^c^

*Note*: Data are mean ± *SD*. See Table 1 for additional abbreviations. Resting and exercise hemodynamics were recorded when the patient was in the semi‐upright position on the cycle ergometer.

Abbreviations: CI, cardiac index; SVi, stroke volume index; WR, work rate.

^a^ *p* < 0.05 vs. rest.

^b^ *p* < 0.05 vs. NOR‐HD.

^c^ *p* < 0.05 vs. Pre‐cap PH.

**FIGURE 1:**
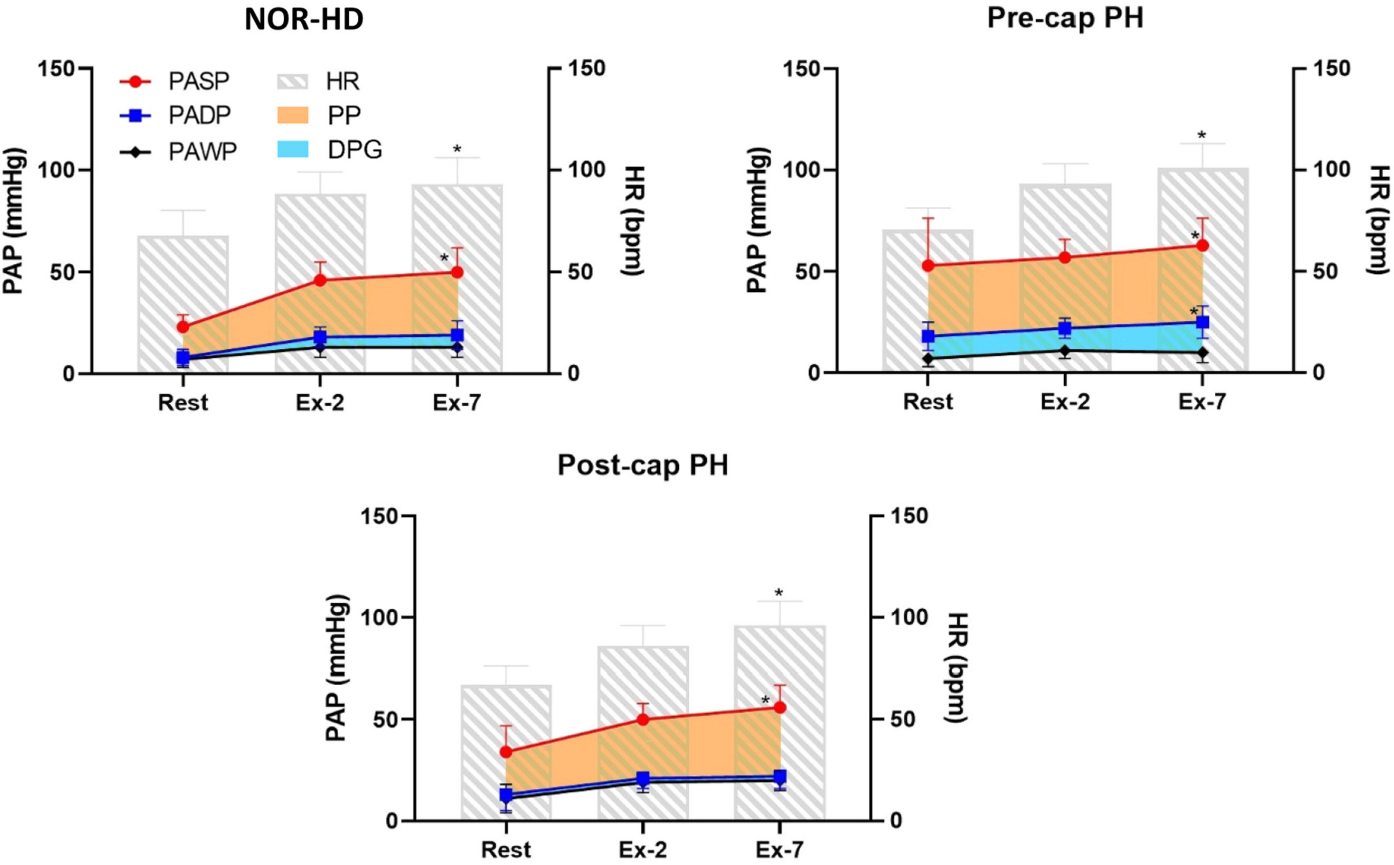
PAWP and pulmonary artery pressures (PAP) at rest, 2 min (Ex‐2) and 7 min (Ex‐7) of exercise in the NOR‐HD, pre‐capillary (pre‐cap) PH, and post‐capillary (post‐cap) PH groups. The orange area represents PP and the blue area represents DPG. *(*p* < 0.05 vs. 2 min). All exercise means are adjusted for age and hemodynamics at rest.

Figure 1 illustrates the pattern of hemodynamic responses at rest, 2 and 7 min of exercise with means adjusted for both age and resting values for variables demonstrating between group differences at rest. HR increased at 2 min and again at 7 min of exercise in all groups. PAWP was higher in the post‐cap PH group but stabilized in all of the groups at 2 min. PADP and DPG did not increase between 2 and 7 min of exercise in the NOR‐HD and post‐cap PH group. By contrast, in the Pre‐cap PH group, PADP and DPG continued to increase at 7 min compared to 2 min of exercise. In all groups, PASP and PP increased significantly at 7 min compared to 2 min of exercise.

### 3.4 Distinguishing features of the resistance‐compliance relationship between pre‐ and post‐cap PH

**FIGURE 2:**
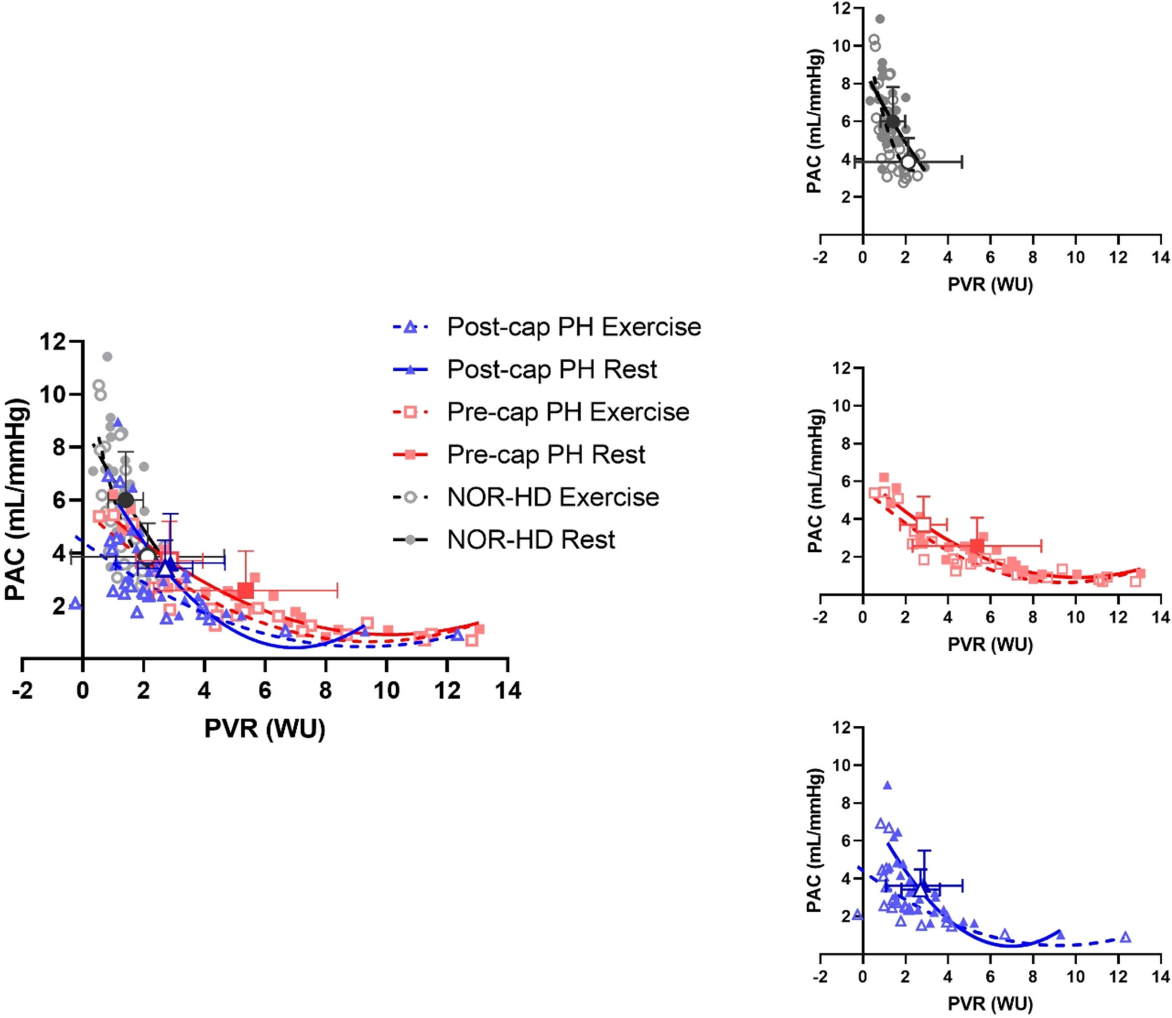
PVR versus PAC graph for rest and exercise in all groups. Large bold symbols represent mean ± *SD* (closed points represent exercise and open points represent rest). Exercise large bold symbols are adjusted for age and hemodynamics at rest. The top right graph represents NOR‐HD, the middle right graph represents pre‐cap PH, the bottom right graph represents post‐cap PH, and the graph on the left represents all groups at rest and exercise.

PVR, PAC, and RC time at rest and exercise are included in Table 4. The relative position of the groups on the resistance‐compliance plot, adjusted for age and resting differences in PAC and PVR, is illustrated in Figure 2. At rest, PVR was highest in the Pre‐cap PH group, intermediate in the post‐cap PH group, and lowest in the NOR‐HD group. With exercise, PVR increased significantly only in the Pre‐cap PH group, persistently remaining the highest. By contrast, PAC declined significantly in all groups during exercise. At rest and exercise, PAC was highest in the NOR‐HD group, while no differences in PAC between pre‐ and post‐cap PH groups were observed.

**FIGURE 3:**
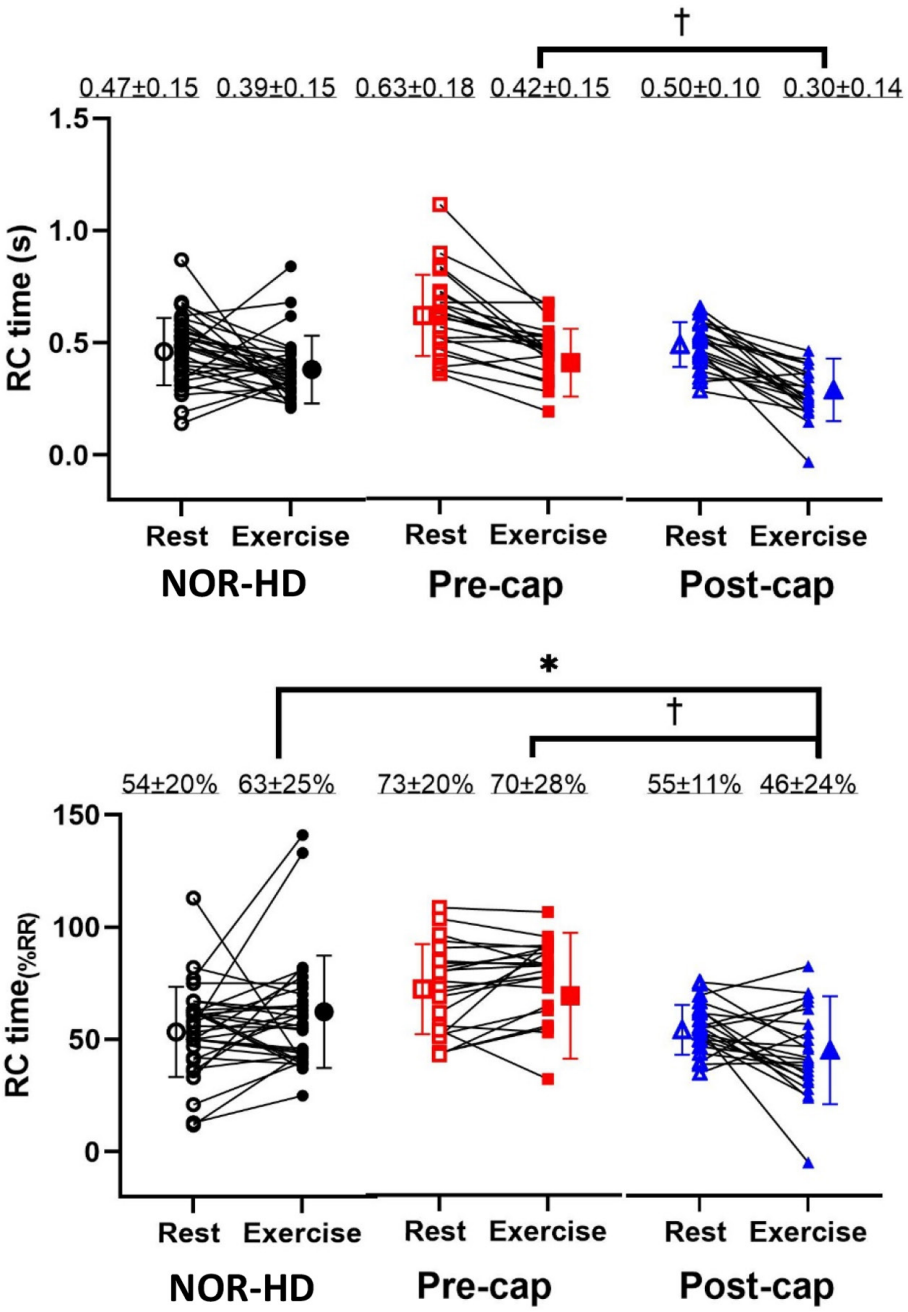
Variations of RC time representations at rest and exercise in all 3 groups. The top graph demonstrates the absolute values of RC time at rest and during exercise in all of the groups. The bottom graph demonstrates RC time as a proportion of the RR interval at rest and exercise in all groups. Larger individual symbols represent the group mean (open symbol represents rest and closed represents exercise). The numerically expressed values at the top of the graphs are group means ± *SD*. Exercise means are adjusted for age and hemodynamics at rest. *(*p* < 0.05 vs. NOR‐HD group during exercise) and †(*p* < 0.05 vs. pre‐cap PH group during exercise).

Figure 3 illustrates the behavior of RC time and RC time_(%RR)_ in all groups at rest and with exercise adjusted for age and resting RC time. At rest, RC time was the longest in the pre‐cap PH group compared to both NOR‐HD and post‐cap PH groups (Table 4). During exercise, the duration of RC time declined significantly in all groups and was shortest in the post‐cap PH group. RC time remained prolonged during exercise in the pre‐cap PH group, though not significantly different compared to the NOR‐HD group.

Expressed as RC time_(%RR)_, this value remained the highest in the pre‐cap PH group. Although exercise shortened RC time, expressed RC time_(%RR)_ did not change significantly in the NOR‐HD or pre‐cap PH group. In contrast, RC time_(%RR)_ declined significantly from rest to exercise (44 ± 20%) in the post‐cap PH group; significantly lower than either the NOR‐HD (60 ± 25%) or the pre‐cap PH group (78 ± 18%).

### 3.5 Associations between hemodynamic variables and changes in RC time during exercise

**FIGURE 4:**
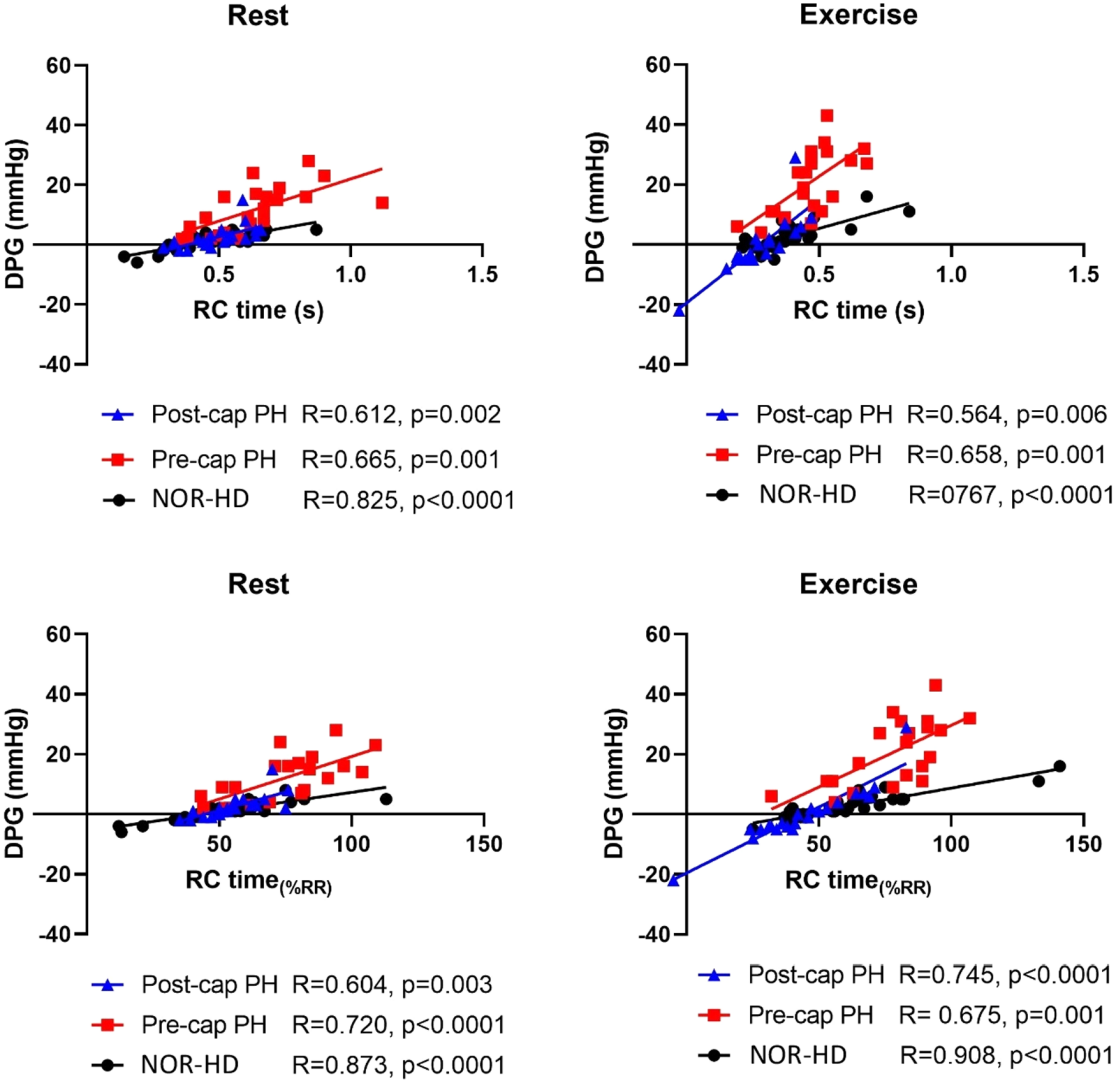
DPG vs. RC time and RC time_(%RR)_ for all groups at rest and exercise.

RC time was not correlated with PAWP, SV, HR, or CO at rest or exercise across all groups (Table 5). In contrast, RC time was correlated with DPG at rest and with exercise in all groups (Figure 4). For the entire population, a multivariable analysis incorporating CO, HR, PAWP, DPG, PADP, and PASP demonstrated that at rest and with exercise RC time was independently highly significantly associated with DPG (Table 6).

**TABLE 5:** Hemodynamic correlations with RC time

	Rest	CO	HR	PAWP	SV	DPG	PADP	PP	TPG	PASP
**NOR‐HD**										
RC time	Pearson's *R*	0.085	0.178	−0.225	−0.187	0.825	0.459	−0.221	0.702	0.121
	*p*‐value	0.643	0.331	0.215	0.306	**<0.0001**	**0.008**	0.225	**<0.0001**	0.508
	Exercise									
RC time	Pearson's *R*	0.135	0.084	−0.331	−0.041	0.767	0.305	−0.278	0.539	−0.075
	*p*‐value	0.460	0.648	0.064	0.822	**<0.0001**	0.089	0.123	**0.001**	0.684
**Pre‐cap PH**										
RC time	Pearson's *R*	−0.231	−0.305	−0.645	−0.125	0.665	0.367	0.005	0.449	0.111
	*p*‐value	0.301	0.168	**0.001**	0.580	**0.001**	0.092	0.983	0.036	0.622
	Exercise									
RC time	Pearson's *R*	−0.468	−0.474	−0.376	−0.372	0.658	0.522	0.374	0.580	0.461
	*p*‐value	**0.028**	**0.026**	0.085	0.089	**0.001**	**0.008**	0.086	**0.005**	**0.031**
**Post‐cap PH**										
RC time	Pearson's *R*	0.080	−0.414	0.072	0.233	0.612	0.352	−0.007	0.302	0.200
	*p*‐value	0.724	0.055	0.750	0.297	**0.002**	0.108	0.974	0.172	0.373
	Exercise									
RC time	Pearson's *R*	−0.207	0.032	−0.395	−0.173	0.564	0.428	0.064	0.626	0.265
	*p*‐value	0.355	0.887	0.068	0.441	**0.006**	**0.047**	0.779	**0.002**	0.234

Bold values indicate *p*‐value <0.05.

**TABLE 6:** Multivariate analysis for the entire population with RC time as the fixed factor

	Rest	CO	HR	PAWP	DPG	PADP	PASP
RC time	*R* squared	0.521	0.708	0.477	0.843	0.604	0.587
	Adjusted *R* squared	−0.123	0.315	−0.226	0.631	0.072	0.033
	*p*‐value	0.744	0.043	0.883	**<0.0001**	0.359	0.438
	Exercise						
RC time	*R* squared	0.581	0.428	0.637	0.760	0.607	0.551
	Adjusted *R* squared	0.173	−0.128	0.283	0.527	0.224	0.113
	*p*‐value	0.142	0.768	**0.038**	**<0.0001**	0.082	0.241

Bold values indicate *p*‐value <0.05.

## 4 DISCUSSION

In this study, we described patterns of pulmonary vascular hemodynamics, and the relationship between pulsatile (PAC) and resistive (PVR) afterload over several minutes of exercise at a modest intensity. We compared these responses among patients with pre‐capillary or post‐capillary PH during exercise compared to patients without exercise PH, adjusting for differences in baseline hemodynamics. Early after exercise onset, PAWP increased but remained stable as exercise continued even in patients with post‐capillary PH. On the other hand, as exercise continued for several minutes, in patients with pre‐capillary PH ongoing changes in PADP and DPG were observed. All groups had a decline in RC time and PAC relative to PVR. However, the dynamics of RC time, expressed as an absolute value or as a proportion of the RR interval was strongly associated with DPG, and were clearly different between pre‐ and post‐capillary PH. Patients with pre‐capillary PH demonstrated the most prolonged RC time related to ongoing increases in DPG despite steady exercise. In contrast, patients with post‐capillary PH demonstrated significant shortening as well as the briefest, exercise RC time. As such, despite large increases of the PAWP in post‐capillary PH, sufficient shortening of RC time appeared to be directly associated with limited increases and actual declines in DPG during exercise, compared to both pre‐capillary PH and NOR‐HD groups.

### 4.1 PAWP remains stable during exercise in pre‐ and post‐capillary states of PH

We previously showed that PAWP increases early after exercise onset in older healthy adults, but does not continue to increase if exercise continues at a constant work rate (Wright, Esfandiari, et al., 2016). We extended our considerations to patients with pre‐capillary and post‐capillary PH, adjusting for age which as expected was higher in the post‐capillary PH group. Although the values for PAWP differed as expected, the pattern of early increases followed by a plateau was confirmed in both PH groups. Our findings are consistent with other studies in patients with heart failure with preserved ejection fraction (Borlaug et al., 2010) and older healthy adults (Kovacs et al., 2009), which have shown the peak magnitude of exercise‐associated increases in PAWP occur early after exercise onset. The temporal pattern of PAWP responses to exercise has not been extensively reported in pre‐capillary PH. Effectively, over the spectrum of pre‐ and post‐capillary PH patients we studied, outflow pulmonary venous pressure, and presumably left ventricular filling pressures, remained stable over a period of constant work‐rate exercise.

### 4.2 Differences in pulmonary vascular hemodynamic patterns during exercise in pre‐ versus post‐capillary PH

In healthy subjects, we have previously demonstrated that PAPs were highly linearly related to the value of PAWP (Wright, Granton, et al., 2016). The slope of the relationship between the PADP and PAWP was approximately 1:1, such that a minimal DPG was observed over a wide range of PAWP and CO responses (Wright, Granton, et al., 2016). Our current study demonstrates that in the pre‐capillary PH group, PADP continued to increase even though both work rate and PAWP remained constant over several minutes of exercise. In all groups, PASP also continued to increase, and as such, PAPs respond to exercise dynamically relative to the pulmonary venous pressure even in the absence of PH. Increases in PASP and pulmonary PP were reflective of decreases in PAC relative to PVR in all groups, in other words, exercise appeared to augment pulsatile load, concordant with our previous findings in healthy subjects (Wright, Granton, et al., 2016).

### 4.3 The duration of RC time in pre‐ and post‐capillary states of PH

The product of PAC and PVR yields a value referred to as RC time which describes a characteristic inverse hyperbolic relationship (Tedford et al., 2012) between these values. The value of RC time also represents a modifiable physiologic characteristic related to the diastolic duration for PAP decay to occur (Chemla et al., 2015). Previous studies have demonstrated that RC time shortens as HR increases (Chemla et al., 2015; Chung et al., 2004; Metkus et al., 2016), which might be an advantageous adaptation to allow pressure decay to occur as the diastolic period decreases.

**FIGURE 5:**
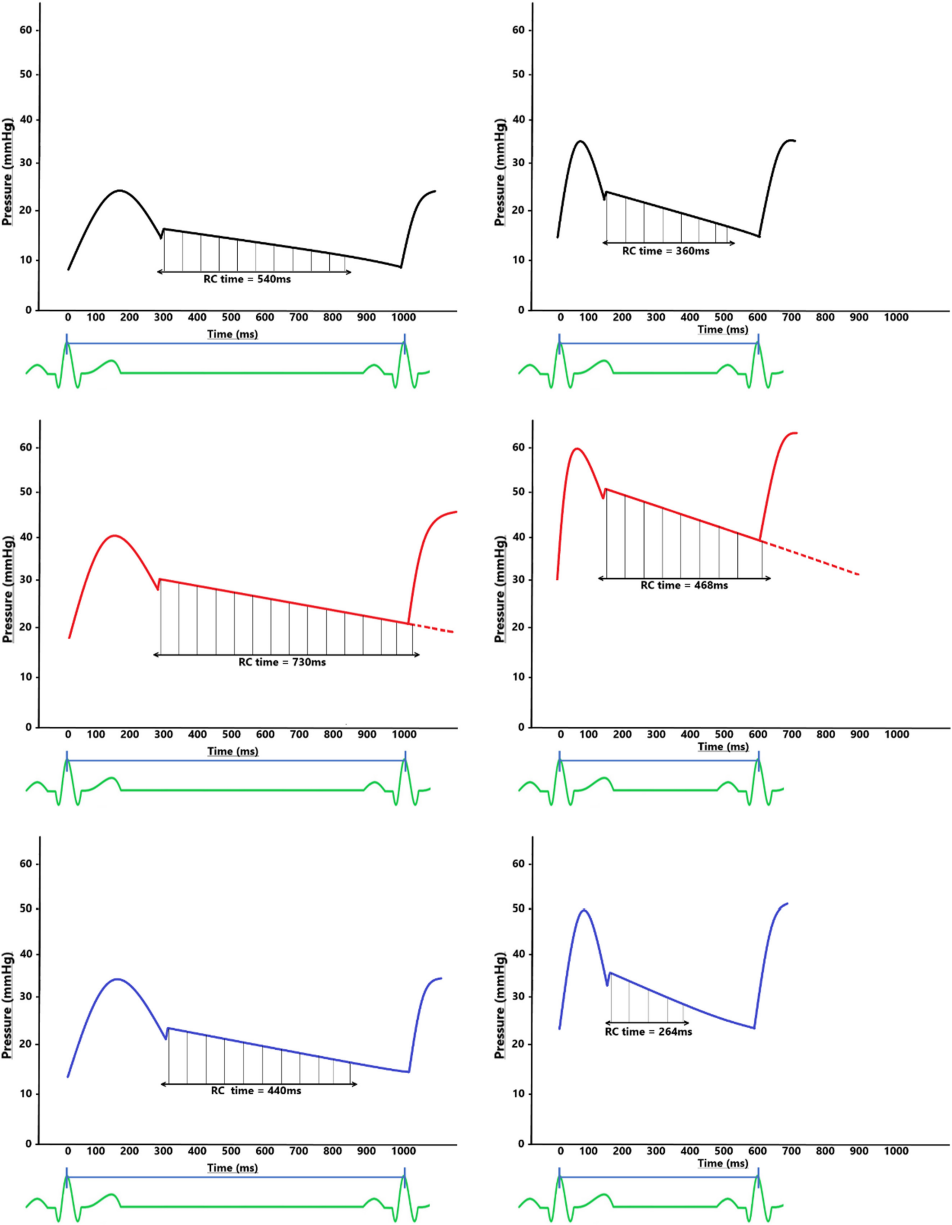
Graphical representation of the impact of the relative length of RC time influencing the temporal pattern of the pulmonary vascular behavior in all groups. The left side represents pressure waveforms at rest with a heart rate of 60 bpm. The right side represents pressure waveforms during exercise with a heart rate of 100 bpm. The top graphs represent the NOR‐HD group, the middle graphs represent the pre‐cap PH group, and the bottom graphs represent the post‐cap PH group.

In the current study, we observed that the duration of RC time and the effect of exercise to shorten it may be distinctive between pre‐capillary and post‐capillary PH. RC time and RC time_(%RR)_ were most prolonged in the pre‐capillary PH group consistent with previous reports (Assad et al., 2016). We have extended these findings to show that RC time remains more prolonged during exercise in pre‐capillary PH and that the duration of RC time is strongly related to the divergence of PADP and PAWP (and thus increasing DPG). This effect to promote increases in DPG and PADP accounted, at least in part, for the greater magnitude and dynamic change in pulmonary pressures observed in the pre‐capillary PH group as exercise continued over several minutes (Figure 5).

In the post‐capillary PH group, the magnitude of PAWP response was the largest, while RC time was the shortest. In addition, there was a decline in DPG with exercise, in sharp contrast to the pre‐capillary group as discussed above. Negative DPG values have been reported at rest in patients with left heart failure; in heart failure, we have previously demonstrated that higher PAWPs are associated with a negative DPG (Tampakakis et al., 2015; Wright et al., 2017). It appears that adequate shortening of RC time (and the more rapid decay of PADP) in the context of increases in HR or PAWP mediates a minimal or even negative DPG during exercise, which in turn limits excessive increases in PAP (Wright et al., 2021). We speculate that the short duration of RC time in the post‐capillary PH group may indicate the preservation of an adaptive physiologic property of the pulmonary vasculature (Figure 5). In pre‐capillary PH, the response to constant work‐rate exercise of an increase in DPG and marked increases in PAP suggests loss of this characteristic of the pulmonary vasculature in this group.

### 4.4 Clinical implication

Our observations have some implications for the conduct and interpretation of exercise hemodynamic testing, which still lacks standardization in methodology (Maron et al., 2020). Exercise RHC may be undertaken to elicit abnormal PAP in patients with dyspnea and risk factors for pulmonary arterial hypertension (PAH) or PH due to left heart disease (LHD; Lau et al., 2015; Maron et al., 2020). Our results suggest that abnormal hemodynamic characteristics are detectable during exercise at a modest effort. Even at a constant work rate, abnormal PAP responses among patients with pre‐capillary PH become increasingly evident as exercise continues over minutes. We confirmed that abnormal PAWP responses in post‐capillary PH were measurable early after exercise onset. Among a population of patients with severe exertional symptoms, compared to a symptom‐limited ramp protocol, a submaximal exercise challenge may improve tolerability and reduce artifacts in hemodynamic tracings induced by respiratory swings. Our insights regarding patterns of exercise responses may further refine clinicians' ability to differentiate between PAH and PH‐LHD, obviously a crucial distinction to be made when selecting candidates for PAH therapies (Charalampopoulos et al., 2018).

### 4.5 Limitations

There are limitations to our study that merit discussion. This is a retrospective and single‐center study. As well, multiple CO measurements by thermodilution were not feasible within an exercise stage, therefore repeated measures of PAC and PVR during exercise were not assessed. Additionally, the need to externally validate the physiological principles demonstrated in this study and show that the results are generalizable will need to be investigated in the future. We acknowledge that the Pre‐cap PH group was heterogeneous, with a variety of co‐morbidities that may be associated with different pathologies within the pulmonary vasculature. Moreover, the estimation of PAC using the SV/PA PP method has been reported previously (Chemla et al., 2015), though there are limitations with the use of fluid‐filled catheters to assess PA PP. However, fluid‐filled catheters remain a pragmatic method to gather sample sizes in the range of this study.

## 5 CONCLUSION

In this study, we demonstrated that the temporal patterns of pulmonary vascular response over several minutes of exercise were distinctly based on the presence and mechanism of PH. Pulmonary vascular afterload continues to change in Pre‐capillary PH as DPG and PADP continue to increase with exercise, related to the prolonged duration of the RC time product. On the other hand, in patients with post‐capillary PH, the shortening of RC time appears to mitigate this behavior.

## CONFLICT OF INTEREST

The authors have no conflicts of interest to disclose.

## ETHICS STATEMENT

This study was approved by the Mount Sinai Research Ethics Board (REB16‐0217‐E).
